# Migration mechanism of a GaN bicrystalline grain boundary as a model system

**DOI:** 10.1038/srep26493

**Published:** 2016-05-23

**Authors:** Sung Bo Lee, Seung Jo Yoo, Young-Min Kim, Jin-Gyu Kim, Heung Nam Han

**Affiliations:** 1 Department of Materials Science and Engineering and Research Institute of Advanced Materials (RIAM), Seoul National University, Seoul 08826, South Korea; 2 Nano-Bio Electron Microscopy Research Group, Korea Basic Science Institute, Daejeon 34133, South Korea; 3 IBS Center for Integrated Nanostructure Physics (CINAP), Institute for Basic Science, Sungkyunkwan University, Suwon 16419, South Korea; 4 Department of Energy Science, Sungkyunkwan University, Suwon 16419, South Korea

## Abstract

Using *in situ* high-resolution transmission electron microscopy, we have explored migration mechanism of a grain boundary in a GaN bicrystal as a model system. During annealing at 500 °C, the grain-boundary region underwent a decrease in thickness, which occurred by decomposition or sublimation of GaN during annealing at 500 °C coupled with electron-beam sputtering. The decrease in thickness corresponds to an increase in the driving force for migration, because the migration of the grain boundary was driven by the surface energy difference. As the driving force increased with annealing time, the grain-boundary morphology turned from atomically smooth to rough, which is characterized by kinetic roughening. The observations indicate that a grain boundary exhibits a *nonlinear* relationship between driving force for migration and migration velocity, in discord with the general presumption that a grain boundary follows a linear relationship.

A number of practical applications ranging from heat treatments of metal and ceramic polycrystals to the fabrication of nanocrystalline materials require the control of grain-boundary (GB) migration. Therefore, understanding of GB migration and structural transitions during its migration is crucial. The aim of the work is to elucidate the point.

As is well documented, crystalline surfaces undergo thermal roughening transitions, which has been theoretically analyzed^1^,^2^ and experimentally verified^3^,^4^. At a critical temperature and above, the free energy required to form a step on a crystalline terrace surface (step free energy, or two-dimensional (2D) nucleation barrier) goes to zero and the flat surface becomes curved, revealing rough atomic structures. It is understood that the flat planes correspond to singular points (or cusps) in the polar plot of the surface energy against the normal direction (γ-plot^5^)^6^. As temperature increases, the step free energy, 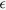, decreases, and goes to zero at a critical temperature, leading to the disappearance of cusps in the γ-plot. This defines a thermal roughening transition and the critical temperature is designated as the thermal roughening transition temperature (*T*_R_). As for crystalline surfaces, GBs are also observed to undergo thermal roughening transitions^7^,^8^.

Besides depending on temperature, the structure of a crystalline surface is also controlled by the driving force for growth, and even below its *T*_R_, it can become roughened at sufficiently high driving forces for growth (kinetic roughening)^9^,^10^,^11^,^12^,^13^,^14^. Kinetic roughening has been well identified in crystal-melt^15^,^16^,^17^,^18^,^19^ and crystal-vapor interfaces^20^. However, although the GB structure is strongly expected to vary with the driving force for migration as in the crystalline surfaces, unambiguous and comprehensive information regarding the atomic process of kinetic roughening in a GB has been missing.

For GBs, just an indication of kinetic roughening was shown for a ZnO bicrystal^21^. However, a detailed knowledge of the GB motion could not be acquired, because the ZnO specimen tended to be severely damaged by electron beams at the high temperature, undergoing amorphisation, and thus *in situ* observation was not possible.

In the present study, using *in situ* high-resolution transmission electron microscopy (HRTEM) at 500 °C of a *GaN bicrystalline GB* as a model material, we have successfully captured a detailed kinetic roughening process in a GB region at the atomic scale. This was possible, because the driving force for GB migration was being increased during observation at 500 °C and we could observe a change in migration behavior with increasing driving force for migration. As annealing progressed at 500 °C, the GB region underwent a decrease in its thickness. The thickness reduction seemed to occur by decomposition or sublimation of GaN in combination with electron-beam sputtering. At such high temperature as 500 °C, the atomic diffusion process at the surface is likely to be dominant, which is doubled by the fact that the TEM specimen examined was measured to be as thin as ~30 nm (see below). Therefore, the GB migration is considered to be driven by the surface energy difference of the two grains meeting at the GB, and the decrease in thickness corresponds to an increase in the driving force for migration. As the driving force increased (i.e., the GB region was thinned), the GB region underwent a morphological change from atomically smooth to undulating, which resulted in a transition in migration behavior from stepwise to continuous, supporting the notion of kinetic roughening of GBs. As will be discussed below, the atomistic observations well prove that GBs exhibit a nonlinear relationship between driving force for migration and migration velocity, in contrast to the general notion that the GB migration velocity is linear with the driving force for migration^22^.

## Results

**Figure 1:**
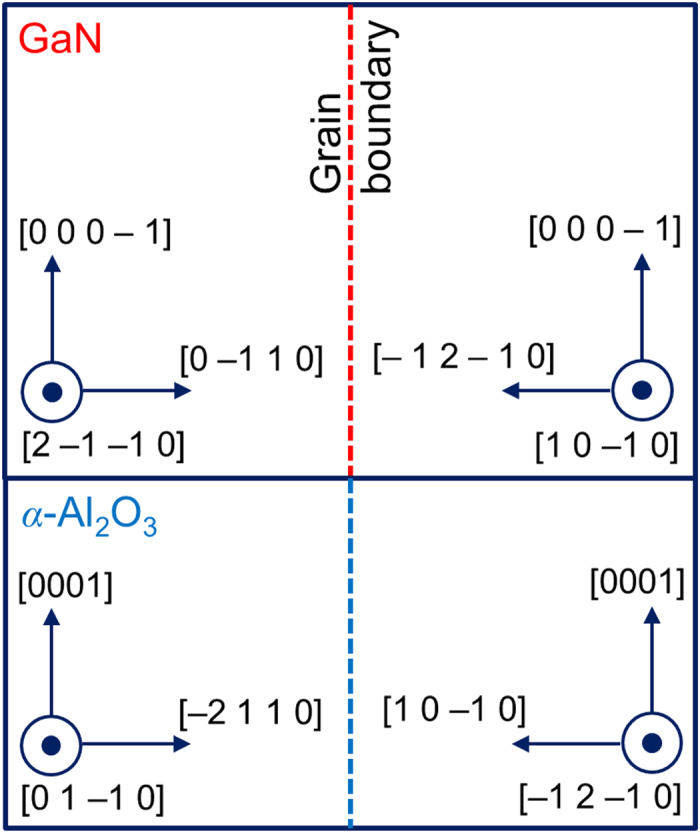
Fabrication of a GaN bicrystal. Cross-section schematic diagram of a GaN bicrystal deposited on the *α*-Al_2_O_3_ bicrystal substrate.

**Figure 2:**
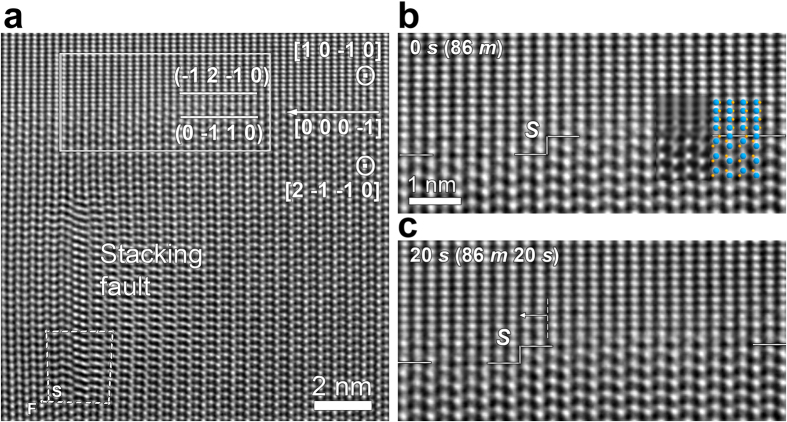
Step migration of the bicrystalline GB. (**a**) Low-magnification overview taken after annealing at 500 °C for 86 min of the GB region between the grains with the surface normal directions of [2 −1 −1 0] and [1 0 −1 0]. A basal-plane stacking fault is seen below the GB, which is used as a gauge of the step and GB migration distances throughout the present study. The [0001] direction indicated by the white arrow is common to both the grains. (**b**) Enlarged HRTEM image of the GB as in (**a)**. The inset is a simulated HRTEM image obtained by employing the multislice method within the MacTempas software package^23^ at a defocus of 16 nm and a specimen thickness of 23 nm based on an atomic model. The atomic positions in the model (blue for gallium and yellow for nitrogen) were determined by contrast matching between the experimental image and simulated images at different defocus and specimen thickness values. For more details on the determination of the GB position, see Supplementary Information (Fig. S1). (**c**) is taken 20 s after (**b**).

Figure 1 is a schematic diagram showing the cross-section view of a GaN bicrystal deposited on an *α*-Al_2_O_3_ bicrystal substrate. (See Methods below for details.) Figure 2a shows the TEM images of a GB region taken after annealing for 86 min at 500 °C. The directions indicated in Fig. 2a are equivalent to those obtained by rotating the schematic diagram (Fig. 1) 90° counterclockwise. The boxed GB region was examined throughout in the present study. The GB typically revealed atomically sharp regions separated by atomic steps with a height of ~0.28 nm roughly, which corresponds to the interplanar spacing of GaN(0 −1 1 0) [0.276 nm] (Fig. 2b). The inset to Fig. 2b shows an HRTEM multislice image simulation^23^ and the underlying rigid atomic model, which determined the position of the GB, as indicated by white horizontal lines (Fig. 2b). (See Supplementary Information (Fig. S1) for more details on the determination of the GB position by the image simulation.) Hereafter, the elapsed time from Fig. 2b as a reference zero will be labeled in the upper left corner of each figure. A time in parenthesis refers to the annealing time at 500 °C for each figure. (Minutes and seconds are abbreviated as *m* and *s*, respectively.) After 20 s from Fig. 2b, the bottom grain with the surface normal direction of [2 −1 −1 0] (grain **1**) grew into the upper grain (grain **2**) by the lateral movement of an atomic step (Fig. 2c) (designated as *S* in Fig. 2b,c). The step advanced to the left from its initial position in Fig. 2b (indicated by a vertical dashed line in Fig. 2c) by ~0.52 nm (corresponding to the interplanar spacing of GaN(0001)) (Fig. 2c). The migration distance is gauged on the image by measuring the distance from the step to a basal-plane stacking fault seen below the boundary (Fig. 2a). The fault is an extrinsic stacking fault^24^. This defect is bounded by two Frank partial dislocations. The Burgers circuit is drawn around the dislocation line of one of the partial dislocations. Its Burgers vector is 1/2 [0 0 0 1]. The (−2 1 1 0) projection of the Burgers vector S–F corresponds to SF/RH (start–finish/righthand) convention. The position of the defect remained unchanged, while the step moved.

**Figure 3:**
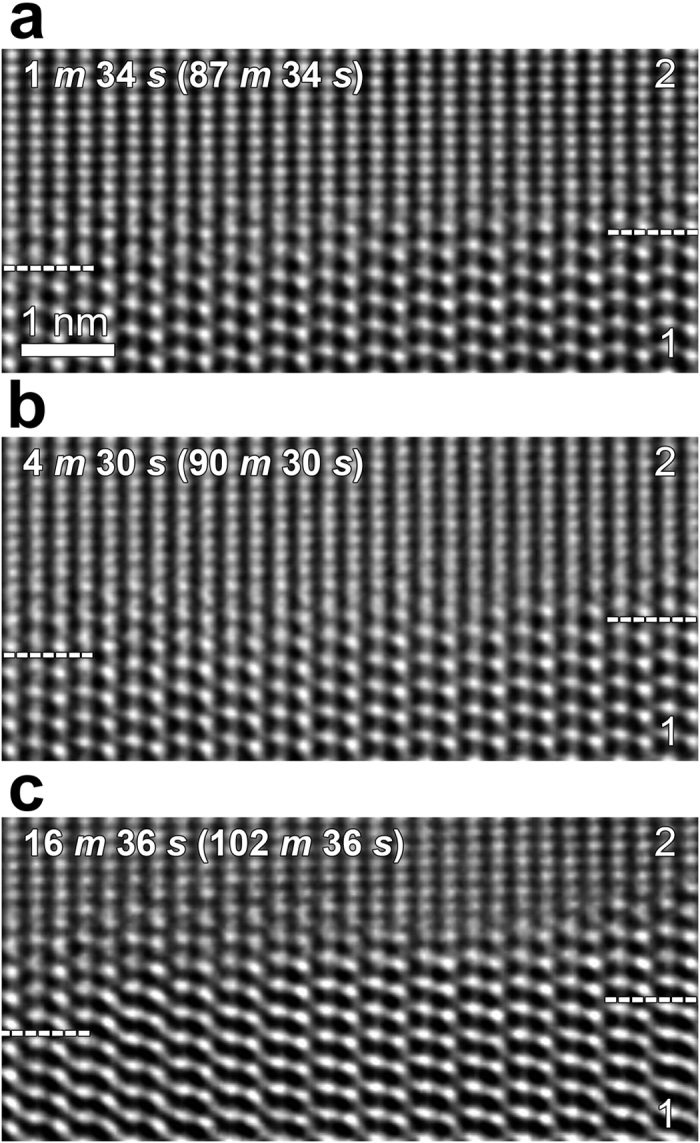
GB roughening after prolonged annealing. A sequence of HRTEM images taken (**a**) 1 min 34 s, (**b**) 4 min 30 s, and (**c**) 16 min 36 s after Fig. 2b. The horizontal dashed lines in the figures mark the initial position of the GB shown in Fig. 2b.

Interestingly, after prolonged annealing times, the GB was no longer sharp, different from the case of the shorter annealing times (Fig. 2). As shown in Fig. 3, it was difficult to define the GB plane orientation, because the GB plane became diffuse and a little wavy. The GB plane was not edge-on any longer (i.e., not parallel to the beam direction or following the initial sharp GB plane bounded by the (0 −1 1 0) and (−1 2 −1 0) planes of the two adjoining grains) and the two grains started to be superposed in the GB region in the beam direction, illuminating that the GB became rough. As indicated by dashed lines marking the initial position of the GB shown in Fig. 2b, grain **1** encroached on grain **2**, and the GB migration did not require atomic steps any longer, but occurred by a *continuous* mechanism (Fig. 3). The rough morphology of the GB became more distinct with increasing annealing time as shown in Fig. 3b,c. The position of the dislocation was not changed during the elapsed time between Figs 2b and 3c, and thus we can easily identify the initial GB position.

Electron energy loss spectroscopy (EELS) was used to examine the possible change in thickness of the *same* GB region before and after annealing at 500 °C. The local thickness of the sample (*t*) was determined using the standard log-ratio formula [*t* = *λ* ln (*I*_t_/*I*_0_)]^25^, where *I*_0_ and *I*_t_ are the areas under the zero loss peak and the entire spectrum (total) in the EELS plot, respectively, and *λ* is the inelastic mean free path for GaN (189.7 nm, calculated with the acceleration voltage of 1250 keV and the collection semiangle of 6.7 mrad). The ln (*I*_t_/*I*_0_) term was calculated to be 0.164 before annealing and 0.13 after annealing, corresponding to a thickness of 31.1 and 24.7 nm, respectively. The thickness range of 31.1−24.7 nm measured by EELS log-ratio method is consistent with the thickness value of 23 nm estimated by the image simulation using Fig. 2a.

## Discussion

The GB migration in the present study is considered to be driven by the difference (Δ*γ*) in surface energy between the two grains, which is given by 2 Δ*γ*/*t*, where *t* is the specimen thickness. According to Northrup and Neugebauer^26^, the GaN(1 0 –1 0) surface is calculated to have a lower energy than the (2 −1 −1 0) surface (118 vs 123 meV Å^−2^; 1 meV Å^−2^ = ~0.016 J m^−2^), which implies that the difference in surface energy between the two orientations is little. Our observation shows that even the (1 1 –2 0) grew into the (1 0 –1 0) grain, in disagreement with the calculation by Northrup and Neugebauer^26^. Taking into consideration both the calculation result that the two surface energies are comparable to each other^26^ and the experimental observation that the (2 −1 −1 0) grain even grew by consuming the (1 0 –1 0) grain, we can assume that even the (2 −1 −1 0) surface has a lower surface energy than the (1 0 –1 0) surface by *a few* meV Å^−2^. Setting the specimen thickness to 30 nm for convenience’s sake, the surface energy difference corresponds to a driving force for migration of a few MPa.

The thickness measurements at the GB region by EELS log-ratio method before and after annealing at 500 °C clearly indicate that the specimen was being thinned during annealing, which is attributed to occur by decomposition of GaN during annealing in vacuum. Mechanisms for GaN decomposition include decomposition into Ga(*g*) and nitrogen, decomposition into Ga(*l*) and nitrogen, or sublimation of GaN as a diatomic or polymeric product [GaN(*g*) or [GaN]_*x*_(*g*)]^27^,^28^, all of which have been experimentally confirmed, as compiled by Koleske *et al.*^28^. However, Ga accumulation on the surface in the form of liquid droplets was not observed during annealing at 500 °C, indicating that the thickness reduction in the present study relies on decomposition into Ga(*g*) and nitrogen, or sublimation.

Since the specimen was irradiated by the high-energy electrons during the observations, the thickness reduction might be influenced by damage due to inelastic (electron−electron) scattering, such as local beam heating and radiolysis, and damage due to elastic (electron−nucleus) scattering, such as knock-on damage and electron-beam sputtering^29^. Electron-beam heating mainly depends on the specimen parameters, such as thermal conductivity and average electron energy loss, and the instrument parameters, such as electron current^29^. Because of the high thermal conductivity of GaN (230 W m^−1^ K^−1^ at 300 K^30^), the temperature rise is calculated to be negligible (ΔT = ~0.01 K). Radiolysis would be also insignificant, considering its relative permittivity and electrical resistivity (see for details Supplementary Information).

Knock-on damage means the direct displacement of atoms *within* a specimen, which occurs only above the threshold incident electron energy and produces point defects. If atomic displacement occurs at the surface of a specimen, it is referred to as sputtering, which is generally known to occur at much lower energies than the determined threshold energy values^29^. According to Look *et al.*^31^, the displacement energies of Ga and N in GaN are 20.5 eV and 10.8 eV, respectively. These values correspond to the threshold energies for displacement^29^ of 450 keV for Ga and 65 keV for N, which are lower than the incident energy of 1250 keV, a circumstance that increases the probability of knock-on damage.

**Figure 4:**
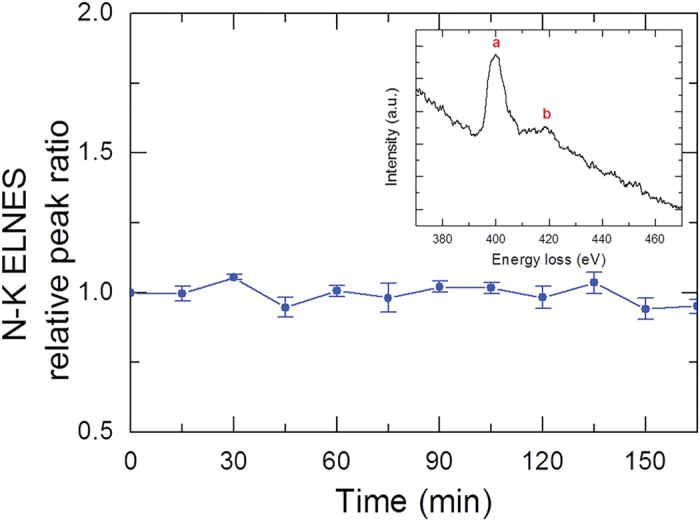
EELS to examine beam damage. Time evolution of N-K ELNES obtained from the GB region. N-K ELNES was acquired every 15 min during annealing for up to 165 min under the same condition as for the *in situ* observation.

However, we did not catch any indication of beam damage possibly formed by knock-on damage in the HRTEM images. To confirm it, we examined a time evolution of energy-loss near-edge structure (ELNES) for nitrogen-K edge from a GB region. The GB region was illuminated under the same condition as for the *in situ* observation (see Methods). We acquired N-K ELNES every 15 min (Fig. 4) and measured the ratio of peak **b** to peak **a** in N-K ELNES (Fig. 4, inset). For convenience’s sake, the ratios measured every 15 min were divided by the ratio at 0 min, a normalised value that is designated as relative peak ratio, as indicated in Fig. 4. As demonstrated in Fig. 4, there was no change in ratio observed even after prolonged annealing for up to 165 min. This tendency indicates that knock-on damage occurring during observation at 500 °C was effectively recovered by annealing at the high temperature. *Actually*, knock-on damage itself will not cause thickness reduction, because it displaces atoms from the crystal lattice and just generates point defects.

Instead, electron-beam sputtering can reduce the specimen thickness. By this process, surface atoms are kicked out into the vacuum, thus leading to mass loss and then thickness reduction. Taken all together, the thickness reduction measured in the present study seems to have originated from sublimation or decomposition of GaN at the high temperature coupled with electron-beam sputtering.

As shown in Fig. 2, when the GB was considered thicker, it assumed an atomically smooth morphology with flat regions separated by an atomic step, which is described by a one-dimensional (1D) terrace-step model. It was observed to migrate by the propagation of the atomic step. It is thus plausible that the 2D nucleation mechanism for crystalline surfaces holds for the smooth GB.

For a atomically smooth surface, where a 2D nucleus on the terrace surface is assumed to adopt the form of a circular disk with an atomic height of *h* and a radius of *r*, with a step free energy, 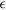
10, the 2D nucleation rate is given by


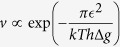


where *k* is the Boltzmann constant, *T* is temperature, Δ*g* is the driving force for nucleation (chemical potential difference between the solid and liquid (or vapor) phases). Below *T*_R_, at small driving forces the surface remains smooth because of the existence of the step free energy (2D nucleation barrier), and its migration occurs by 2D nucleation or to be mediated by growth spirals supplied by screw dislocations (stepwise mechanism)^10^. As expressed by the above equation, in the 2D nucleation regime, the stepwise migration mechanism is characterised by a nonlinear, exponential relationship between migration rate and the driving force^10^. As the driving force for nucleation is increased in the 2D nucleation regime, the rate deviates positively from the rate predicted by the classical 2D nucleation limited growth, which is due to the decrease of the step free energy with increasing the driving force^9^,^11^,^17^.

At a critical driving force and above, the width of a step exceeds the radius of the 2D nucleus^9^,^11^ and the step formed by the 2D nucleus loses its physical entity in the background of the interface^9^,^11^,^17^. It is thus understood that, under such *nonequilibrium* conditions, the step free energy goes to zero and the surface becomes rough^9^,^11^,^13^,^14^,^17^. Under this circumstance, the GB is expected to move by continuous growth (three-dimensional nucleation, or multinuclear growth, as for crystalline surfaces^9^,^10^,^11^,^12^,^13^,^14^,^15^,^16^,^17^,^18^,^19^,^20^), where its growth rate is expected to be linear with Δ*g* because the growth is limited by the atomic flux normal to the interface without any requirement of steps. Taken together, the relationship of migration rate, *v*, with the driving force turns from exponential (for stepwise growth) to linear (for continuous growth) at a critical value of the driving force. Through an investigation of the solidification kinetics of liquid Ga, Peteves and Abbaschian experimentally confirmed the prediction^17^. For GB migration, all terms in the above equation have usual meanings corresponding to a GB, i.e., *h* is the atomic step height on the GB plane, 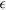 the GB step free energy and Δ*g* the driving force for GB migration.

As illuminated in Figs 2 and 3, with decreasing specimen thickness (in other words, increasing the driving force for migration), the same GB undergoes a morphological change from atomically smooth to rough, accompanied by a transition from stepwise to continuous migration regimes. As shown in Fig. 3, when the GB becomes roughened, the migration was observed to be accelerated. The changes in GB morphology and in migration mechanism (Figs 2 and 3) are evidence of kinetic roughening because they are accompanied by the increase in the driving force for GB migration.

The phenomenon observed in the present study has nothing to do with a thermal roughening transition, because the observed transition does not depend on the driving force for migration. The flat morphology of the GB at the lower driving forces for migration (i.e., when the GB region is thicker) suggests that the thermal roughening transition temperature (*T*_R_) is certainly higher than 500 °C.

Such nonlinear relationship appears in polycrystalline systems. In polycrystalline materials, at low temperatures, most GBs are faceted and abnormal grain growth occurs, and at higher temperatures, GBs become rough and normal grain growth occurs^32^,^33^,^34^. It was suggested that if GBs are faceted, they advance by two-dimensional nucleation at low driving forces for migration, and that at higher driving forces, they advance by a continuous growth mechanism^32^,^33^,^34^. Thus, if most GBs are faceted, only grains with size advantages can grow and abnormal grain growth occurs. If most GBs are already roughened, they are expected to migrate by a continuous growth mode, leading to normal grain growth^32^,^33^,^34^. However, their results required direct evidence of kinetic roughening for a single GB, which is provided by the present study.

Taken together, although a GB is not thermally roughened, an increase in the driving force for migration tends to cause the GB to become rough, i.e., kinetically roughened. A rough GB is characterised by its open structure, which accommodates more defects and impurities than can a singular GB with atomically well-matched structure^35^. Such a concept is not limited to a single GB but is well extended to those in polycrystalline materials, especially nanocrystalline materials. The concept of kinetic roughening well explains why GBs in nanocrystalline materials have higher GB diffusivities and mobilities than those in coarser-grained materials^36^,^37^. GBs in nanocrystalline materials are subjected to higher driving forces for migration than those in polycrystals with larger grain sizes and are thus likely to become kinetically roughened easily at a fixed temperature. Of course, a tendency toward kinetic roughening would be different for GBs with different GB characteristics, such as misorientation relationships and GB plane orientations, an expectation which motivates detailed experiments using bicrystals with various GB characteristics.

Grain boundaries are no exception. The results here certainly provide evidence that GBs also undergo kinetic roughening like other crystalline interfaces, which was identified by *in situ* HRTEM investigations of a model GaN bicrystalline GB. As the driving force for migration increased with increasing annealing time at 500 °C (i.e., as the specimen thickness decreased), the GB underwent a transformation from atomically smooth to rough and wavy shapes. The reduction in the specimen thickness is considered to occur by a coupling of decomposition or sublimation of GaN during annealing at 500 °C and electron-beam sputtering. The morphological change was accompanied by a transition from stepwise to continuous migration mechanisms, corroborating the idea of GB kinetic roughening. As discussed above, kinetic roughening is also generalised to GBs in polycrystalline materials, prompting further study on the relationship between GB characteristics and kinetic roughening.

## Methods

### Fabrication of a bicrystal

A GaN bicrystal was deposited to a thickness of ~3.5 μm by metalorganic chemical vapor deposition on an *α*-Al_2_O_3_ bicrystal substrate (Shinkosha Co., Ltd., Japan). The bicrystal substrate was composed of two grains, both with a surface normal direction of [0001], with a misorientation relationship of [0001]/30°, where the GB was bounded by (−2 1 1 0) and (1 0 −1 0) planes of the two adjoining grains (Fig. 1). Following the epitaxial relationship between GaN and *α*-Al_2_O_3_ [(0001) GaN//(0001) *α*-Al_2_O_3_ and [1 0 −1 0] GaN//[−1 2 −1 0] *α*-Al_2_O_3_], the resultant GaN bicrystalline GB had the same misorientation relationship as the bicrystal substrate, bounded by (0 −1 1 0) and (−1 2 −1 0) planes of the two adjoining grains.

### TEM specimen preparation and *in-situ* observation

TEM specimens were prepared by FIB lift-out technique from the film surface. Cross-section lamellae specimens for *in situ* HRTEM were prepared on a focused Ga-ion beam (FIB) workstation (FEI Nova 200 Nanolab dual-beam FIB). The specimen was composed of two grains with different surface normal directions of [2 −1 −1 0] (left, Fig. 1) and [1 0 −1 0] (right, Fig. 1). The prepared specimen was heated to 500 °C at a heating rate of 20 °C/min in a high-voltage TEM (HVTEM) operating at 1250 keV (0.12 nm point-to-point resolution) (JEOL JEM-ARM1300S, JEOL), which is equipped with a side entry heating stage. Generally, on reaching at elevated temperatures for *in situ* TEM observation, a specimen undergoes thermal instability, such as specimen drift, which hinders us from normal observations. Thus we had to wait for at least 30 min for the specimen to be stabilised after reaching the high temperature. The GB morphology was examined during annealing at 500 °C in the TEM. The base pressure in the specimen chamber of the HVTEM was ~2 × 10^−6^ Pa. The annealing time at 500 °C was ~114 min in total. After annealing at 500 °C, the specimen was cooled to room temperature by turning off the heating stage controller connected to the TEM specimen heating holder, where the cooling rate was ~186 °C/min from 500 °C to 100 °C and ~3.6 °C/min from 100 °C to room temperature. The thickness of the specimen before and after annealing at 500 °C was determined by EELS log-ratio method^25^. The electron dose rate during *in situ* observation was 1.06 × 10^5^ e^−^/nm^2^ s (~1.7 A/cm^2^).

### EELS for nitrogen-K edge

To examine how much beam damage was introduced under such an irradiation condition during *in situ* observation, EELS for N-K edge was additionally performed at 500 °C with a HV-GIF system (Gatan) attached to the aforementioned ARM. The GB region was illuminated under the same condition as for the *in situ* observation. The EELS spectrum was acquired every 15 min during annealing for 165 min at 500 °C. The energy resolution at the zero-loss peak was typically 1.3 eV. Each EELS spectrum was acquired using a 2 mm EELS entrance aperture, which determined the collection semiangle of 3.4 mrad, at a dispersion of 0.2 eV per channel.

## Additional Information

**How to cite this article**: Lee, S. B. *et al.* Migration mechanism of a GaN bicrystalline grain boundary as a model system. *Sci. Rep.* **6**, 26493; doi: 10.1038/srep26493 (2016).

## Supplementary Material

Supplementary Information
