# Supplementary material for: Migration mechanism of a GaN bicrystalline grain boundary as a model system
Source: Sci Rep. 2016 May 23;6:26493. doi: 10.1038/srep26493 (PMC4876465; doi:10.1038/srep26493)
Supplement: Supplementary Information [file srep26493-s1.doc]

**Supplementary Information**

Migration mechanism of a GaN bicrystalline grain boundary as a model system

Sung Bo Lee,^1^,* Seung Jo Yoo,^2^ Young-Min Kim,^3,4^ Jin-Gyu Kim,^2^ and Heung Nam Han^1^

^1^Department of Materials Science and Engineering and Research Institute of Advanced Materials (RIAM), Seoul National University, Seoul 08826, South Korea. ^2^Nano-Bio Electron Microscopy Research Group, Korea Basic Science Institute, Daejeon 34133, South Korea. ^3^IBS Center for Integrated Nanostructure Physics (CINAP), Institute for Basic Science, Sungkyunkwan University, Suwon 16419, South Korea. ^4^Department of Energy Science, Sungkyunkwan University, Suwon 16419, South Korea.

**Comment on the determination of the grain boundary position by the image simulation**

**Fig. S1**

**Comment on the effect of radiolysis on the specimen thickness reduction**

**References**

**Determination of the grain boundary position by the image simulation**. We have defined the grain boundary position with the help of an image simulation^1^. Contrast delocalization due to lens aberrations hampers exactly defining the interface between two conjoined materials since it is expressed as the lateral displacement of spatial frequencies in the image, thus causing a significant imaging artefact in the HRTEM image. In the observation condition of 1250-keV high-voltage electron microscope using a thermionic LaB_6_ electron source that is showing relatively larger source spread parameter than field emission electron source, HRTEM image simulation confirms that the artificial displacement of surface/interface atoms due to the contrast perturbation is estimated to be less than ~2 Å that is much less than the observed interplanar spacings of GaN in the experiment (not shown here). Therefore, we can reliably define the interface for the HRTEM image obtained by the microscope based on the model-based image simulation as shown in **Fig. S1**.


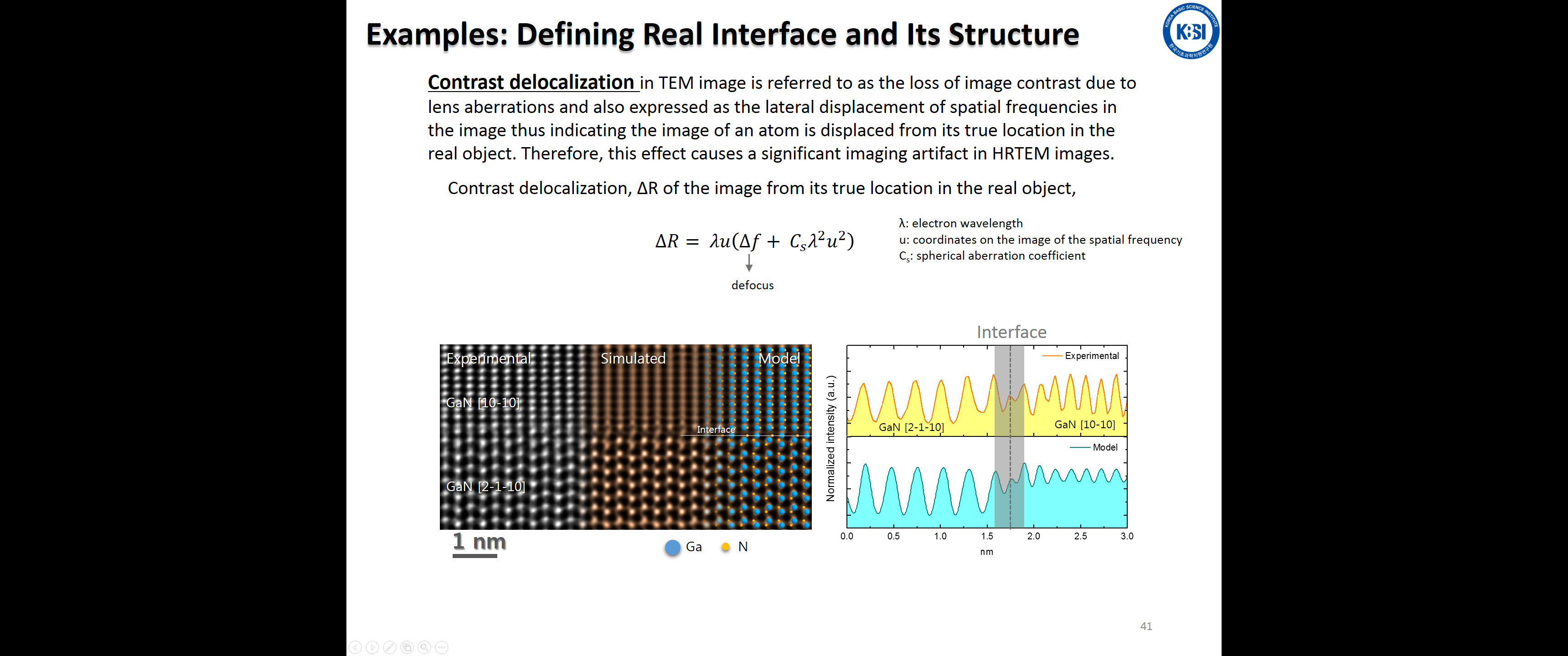


**Fig. S1**

**Effect of radiolysis on the specimen thickness reduction.** Radiolysis indicates chemical bond breaking through inelastic (electron−electron) scattering, sometimes inducing the hole drilling in the specimen^2,3^. For GaN, radiolysis would be insignificant as compared with damage due to elastic scattering. For its occurrence, electronic excitations of atomic electrons by a beam of electrons must remain long enough to cause atomic displacement, for which the relaxation time of excited electrons should be longer than 1 ps^4^. The relaxation time is approximated by^4^ *τ* = *ε*_0_ *ε*_r_ *ρ*_R_, where *ε*_0_, *ε*_r_, and *ρ*_R_ are the vacuum permittivity, the relative permittivity, and the resistivity of a material. However, for conducting materials, whose resistivity is less than 10^−3^−10^−4^ Ω m, electronic excitations are too short to cause atomic displacement and damage due to inelastic scattering would be minor, because the excitations can be rapidly delocalized by the conduction electrons^4^. For undoped GaN, the resistivity is determined to be ~10^−3^ Ω m (for undoped GaN layer on sapphire by vapor phase technique)^5,6^ and the relative permittivity is 8.9, producing a relaxation time of 7.9 × 10^−2^ ps, too short to cause atomic displacement. Thus, both the bulk and the surface of GaN are not likely to be damaged by radiolysis. Actually, our present study does not show any

**References**

1. Kilaas, R. In *Proc. 45th Annual Meeting of EMSA* (ed Bailey, G.W.) 66–69 (Baltimore, Maryland, 1987).

2. Egerton, R.F., Li, P. & Malac, M. Radiation damage in the TEM and SEM. *Micron* **35**, 399−409 (2004).

3. Humphreys, J., Bullough, T. J., Devenish, R. W., Maher, D. M. & Turner, P. S. Electron beam nano-etching in oxides, fluorides, metals and semiconductors. *Scanning Microsc. Suppl.* **4**, 185–192 (1990).

4. Jiang, N. Electron beam damage in oxides: a review. *Rep. Prog. Phys.* **79**, 1−33 (2016).

5. Ilegems, M. Vapor epitaxy of gallium nitride. *J. Cryst. Growth* **13/14**, 360−364 (1972).

6. Crouch, R. K., Debnam, W. J. & Fripp, A. L. Properties of GaN grown on sapphire substrates. *J. Mater. Sci.* **13**, 2358−2364 (1978).
